# Variation in incidence of breast, lung and cervical cancer and malignant melanoma of skin by socioeconomic group in England

**DOI:** 10.1186/1471-2407-8-271

**Published:** 2008-09-26

**Authors:** Lorraine Shack, Catrina Jordan, Catherine S Thomson, Vivian Mak, Henrik Møller, UK Association of Cancer Registries

**Affiliations:** 1 North West Cancer Intelligence Service, Christie Hospital NHS Trust, Kinnaird Road, Withington, Manchester, M20 4QL, UK; 2 Non-communicable Disease and Epidemiology Unit, London School of Hygiene and Tropical Medicine, WC1E 7HT, UK; 3 Trent Cancer Registry, 5 Old Fulwood Road, Sheffield, S10 3TG, UK; 4 Cancer Research UK, London, WC2A 3PX, UK; 5 King's College London, Thames Cancer Registry, 1st Floor, Capital House, 42 Weston Street, London, SE1 3QD, UK

## Abstract

**Background:**

Cancer incidence varies by socioeconomic group and these variations have been linked with environmental and lifestyle factors, differences in access to health care and health seeking behaviour. Socioeconomic variations in cancer incidence by region and age are less clearly understood but they are crucial for targeting prevention measures and health care commissioning.

**Methods:**

Data were obtained from all eight English cancer registries for patients diagnosed between 1998 and 2003, for all invasive cases of female breast cancer (ICD-10 code C50), lung cancer (ICD-10 codes C33-C34), cervical cancer (ICD-10 code C53), and malignant melanoma of the skin (ICD-10 code C43). Socioeconomic status was assigned to each patient based on their postcode of residence at diagnosis, using the income domain of the Index of Multiple Deprivation 2004. We analysed the socioeconomic variations in the incidence of breast, lung and cervical cancer and malignant melanoma of the skin for England, and regionally and by age.

**Results:**

Incidence was highest for the most deprived patients for lung cancer and cervical cancer, whilst the opposite was observed for malignant melanoma and breast cancer. The difference in incidence between the most and the least deprived groups was higher for lung cancer patients aged under 65 at diagnosis than those over 65 at diagnosis, which may indicate a cohort effect. There were regional differences in the socioeconomic gradients with the gap being widest for lung and cervical cancer in the North (North East, North West and Yorkshire and Humberside) and for malignant melanoma in the East and South West. There were only modest variations in breast cancer incidence by region. If the incidence of lung and cervical cancer were decreased to that of the least deprived group it would prevent 36% of lung cancer cases in men, 38% of lung cancer cases in women and 28% of cervical cancer cases. Incidence of breast cancer and melanoma was highest in the least deprived group, therefore if all socioeconomic groups had incidence rates similar to the least deprived group it is estimated that the number of cases would increase by 7% for breast cancer, 27% for melanoma in men and 29% for melanoma in women.

**Conclusion:**

National comparison of socioeconomic variations in cancer incidence by region and age can provide an unbiased basis for public health prevention and health commissioning. Decreasing inequalities in incidence requires the integration of information on risk factors, incidence and projected incidence but targeted public health interventions could help to reduce regional inequalities in incidence and reduce the future cancer burden.

## Background

Incidence for many cancers varies by socioeconomic group in the UK [1-3] and other countries [4-6]. Socioeconomic variations have been attributed to environment [7], lifestyle [8,9], biological effects [10], access to health care [11-17] and health seeking behaviour [18-21]. These variations are particularly wide for the cancers under study (breast, cervix, lung, malignant melanoma) due to the close association between socioeconomic status and risk factors. Inequalities in mortality[22] and survival [5,23-30] have been evaluated to assess improvements in inequities in outcome and access to treatment. National comparison of socioeconomic variations in cancer incidence by region and age are less understood but provide an unbiased basis for prevention and commissioning. Previous studies have focused on regional variations [22,31] with one recent study of regional and socioeconomic variation in breast and lung cancer[32], although this did not include age variations. This study aims to investigate socio-economic differences in breast, lung, cervical and malignant melanoma cancer incidence among patients diagnosed in England during 1998–2003, by region and age.

## Methods

Data were obtained from all eight English cancer registries for patients diagnosed between 1998 and 2003, for all invasive cases of female breast cancer (ICD-10 code C50), lung cancer (ICD-10 codes C33-C34), cervical cancer (ICD-10 code C53), and malignant melanoma of the skin (ICD-10 code C43). These cancer sites were selected due to their association with risk factors closely linked to socioeconomic status, such as smoking, UV exposure, HPV infection and reproductive factors. Breast and cervical cancer were of specific interest due to socioeconomic variations in screening up-take.

A socioeconomic group was assigned to each patient based on their postcode of residence at diagnosis, using the income domain of the Index of Multiple Deprivation 2004 (IMD) [33]. IMD is a national measure of deprivation for each Lower Super Output Area in England based on information collected at census and in government databases, such as income support and job seekers allowance. IMD is based on seven domains of deprivation (income, employment, health deprivation and disability, education skills and training, barriers to housing and services, crime and living environment). To exclude the health related measures of the IMD, only the income domain of the IMD was used in this study. This has been shown to strongly correlated with deprivation and exclude health related measures [25,34]. The national income domain of the IMD for all lower super output areas (LSOAs) in England was ranked in ascending order and divided into quintiles, each containing 20% of the total population of England [35], with quintile 1 being least deprived fifth of the population and quintile 5 the most deprived fifth of population. Super output areas are geographies that were introduced by the Office for National Statistics (ONS) in and attempt to create a uniform geographic system across England for analysis that stays stable over time. There are 32,482 lower super output areas (LSOAs) in England with an average population size of 1,500 residents [36].

**Figure 1:**
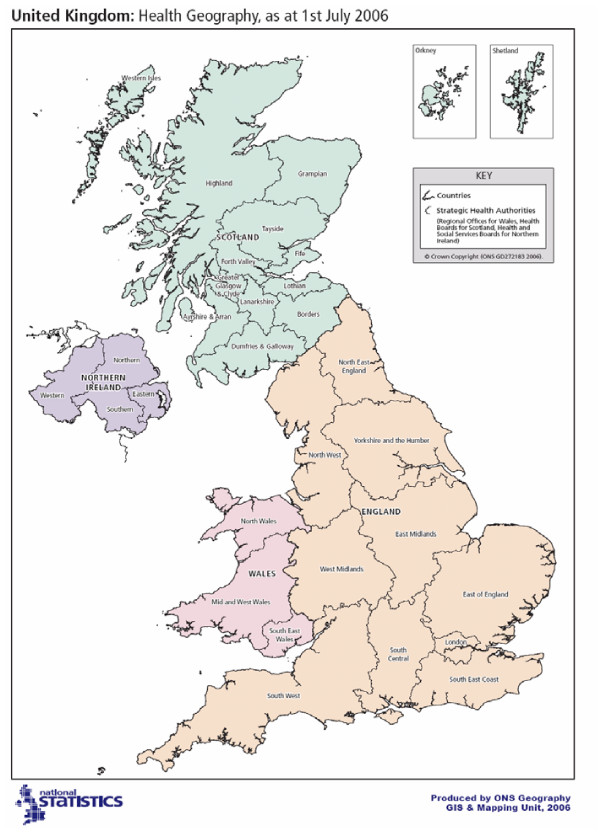
Map of Regions in UK.

European age-standardised incidence rates (ASRs) were calculated by deprivation quintile, cancer site, Government Office Region (GOR) (Figure 1), age group (under 65, 65 and over) and gender. Rate Ratios (RR) were calculated by region and age (under 65 and 65 and over) using the least deprived (quintile 1) as the baseline. The number (and percentage) of additional/fewer incident cases that would be expected if incidence rates in all deprivation groups was the same as those in the least deprived was estimated. The difference between the observed and expected number of cases for socioeconomic group was calculated to provide an estimate of the burden of cancer should these inequalities change. All analyses were done in EXCEL and STATA 8.0 [37].

This study was carried out in association with the United Kingdom association of Cancer Registries (UKACR) which collect and conduct cancer surveillance using the data they collect under Section 60 of the Health and Social Care Act 2002. The study used an anonymised dataset and separate ethical approval was not required.

## Results

The highest incidence rates in England occurred in the most deprived groups for both lung cancer and cervical cancer, while the opposite was the case for malignant melanoma and breast cancer with the least deprived groups having the highest incidence (Table 1). The inequality between the least deprived, and the most deprived was highest for lung cancer in men (RR 2.53 95% CI: 2.48–2.58) and women (RR 2.73 95% CI: 2.66–2.80). Similarly, cervical cancer incidence was highest in the most deprived group (RR 2.08 95% CI: 1.97–2.19) and decreased consistently with increasing affluence. If all population groups has the same incidence rates as the least deprived group, we would expect 41,076 (36%) fewer lung cancer cases in men, 28,148 (38%) fewer cases in women and 4,108 (28%) fewer cervical cancer cases. Breast cancer incidence was highest in the least deprived with modest differences between socioeconomic groups (Breast: RR 0.84 95% CI: 0.82–0.85). Malignant melanoma incidence showed wide variations by socioeconomic status but similar trends for men and women (Melanoma in men: RR 0.49 95% CI: 0.47–0.52, Melanoma in women: RR 0.48 95% CI: 0.46–0.51). If the socioeconomic-specific incidence rates where equal to those in the least deprived quintile across all population groups the number of breast cancer cases would rise by 15,496 (7%) and cases of malignant melanoma of the skin would increase by 4,286 (27%) in men and 5,862 (29%) in women.

**Table 1:** Incidence by socioeconomic status for lung, breast, cervical, and melanoma cancer, England, 1998–2003

Men						Women					
Deprivation	No.	AVR	RR	Excess cases compared to least deprived	%	Deprivation	No.	AVR	RR	Excess cases compared to least deprived	%

Lung						Lung					
Least Deprived	15177	42.2	1.00	-		Least Deprived	9024	21.1	1.00	-	
2	19341	51.1	1.21	-3296		2	12015	25.7	1.22	-2782	
3	22572	52.1	1.46	-7143		3	14286	30.7	1.46	-4504	
4	26125	79.2	1.87	-12,137		4	17246	40.5	1.92	-8281	
Most Deprived	30573	107.4	2.53	-18499		Most Deprived	20789	57.5	2.73	-13180	
Total	113788			-41076	-36.1	Total	73360			-28148	-38.4

Melanoma						Melanoma					
Least Deprived	4239	12.8	1.00	-		Least Deprived	5217	14.8	1.00	-	
2	3908	11.5	0.90	425		2	4901	13.3	0.90	539	
3	3368	10.3	0.81	796		3	4356	11.8	0.80	1099	
4	2571	8.5	0.67	1292		4	3506	9.9	0.67	1715	
Most Deprived	1724	6.3	0.49	1772		Most Deprived	2352	7.2	0.484	2509	

Total	15810			4286		Total	20332			5862	28.8

						Breast					
						
						Least Deprived	46085	125.7	1.00	-	
						2	46745	121.6	0.97	1557	
						3	44718	118.1	0.94	2859	
						4	39800	112.6	0.90	4643	
						Most Deprived	32672	105.0	0.84	2509	
						
						Total	210020				7.4
						
						Cervix					
						
						Least Deprived	2149	6.4	1.00	-	
						2	2515	7.2	1.13	-289	
						3	2782	7.8	1.23	-520	
						4	3362	9.9	1.54	-1185	
						Most Deprived	4072	13.3	2.08	-2113	
						
						Total	14880			-4108	-27.6

**Figure 2:**
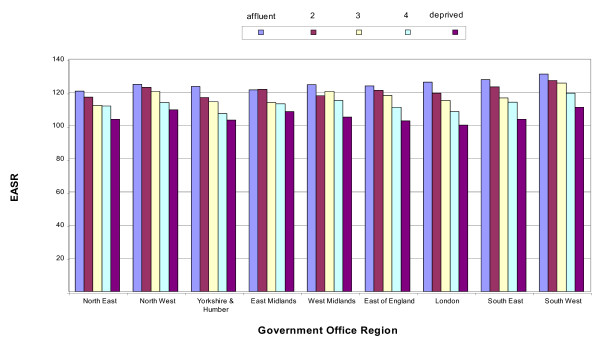
Breast cancer incidence (European age-standardised rate per 100,000) by Government Office Region, England, 1998–2003.

**Figure 3:**
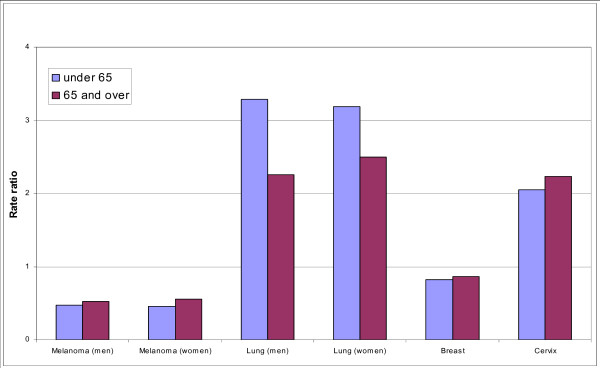
Rate ratio for cancer incidence for patients in least to most deprived socioeconomic group, England, 1998–2003.

There were only modest differences in socioeconomic-specific breast cancer incidence rates both between and within regions, with the highest in the East Midlands (RR 0.89 95% CI 0.85–0.94) and the lowest in London (RR 0.79 95% CI 0.76–0.83) (Figure 2). Socioeconomic variations by age were small and not significantly different for women aged under 65 and over 65 (Figure 3).

Cervical cancer incidence varied substantially within regions by socioeconomic group with a large increase in incidence occurring between the second and the most deprived group in most regions, except London and the East of England. This difference was most significant in the South West with an age standardised incidence rate of 10.3 per 100,000 (95% CI: 9.1–11.5) in the second most deprived and 15.5 per 100,000 (95% CI: 13.3–17.7) in the most deprived group. Socioeconomic-specific incidence rates in general, as well as the difference between the most and least deprived were lowest in London and the East of England. In contrast the highest incidence rates were recorded in deprived groups in the North West and South West. The gap between most and least deprived was greatest in the North West and Yorkshire and Humberside. The deprivation gap was not statistically different between those under 65 and 65 and over.

**Figure 4:**
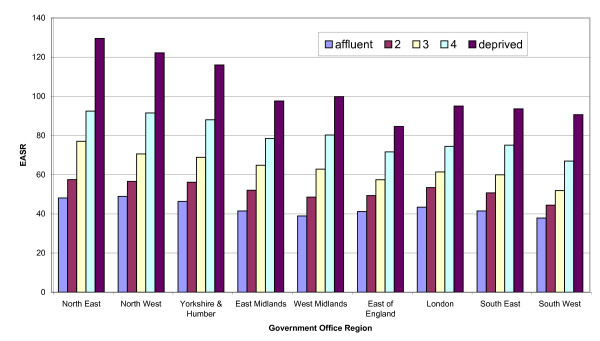
Lung cancer incidence in men (European age-standardised rate per  100,000) by Government Office Region, England, 1998-2003.

Incidence rates for lung cancer were 40% lower in women than men however the trends by age and regional were still similar. The highest incidence rates occurred in the most deprived group for both men and women in the North East and North West, with the lowest occurring in the East of England (Figure 2). The North East and West Midlands had the highest deprivation gap in men, with the North West and North East having the highest for women. Lung cancer was the only site with a significant difference in the deprivation gap by age group with those aged under 65 having a higher gap (men: RR 3.29 95% CI: 3.16–3.41, women: RR 3.18 95% CI 3.03–3.34) than those age 65 and over (men: RR 2.26 95% CI 2.20–2.30 and women: RR 2.29 95% CI 2.42–2.56) (Figure 4).

The deprivation gap for malignant melanoma was similar in men and women, with the exception of the North East where it was larger in men, and the South West, where it is larger in women. Incidence was higher for each socioeconomic group and the deprivation gap narrower in the South West than in other regions, for both men (RR 0.84 95% CI: 0.70–1.00) and women (RR 0.69 95% CI: 0.58–0.81). In the North East there was some inconsistency in socioeconomic-specific incidence for malignant melanoma as incidence in the second least deprived group had lower incidence than the socioeconomic groups on either side. London had the lowest incidence for all deprivation groups, in men and women. The largest difference between least and most deprived occurred in West Midlands for men (RR 0.43 95% CI: 0.36–0.51) and Yorkshire and Humber for women (RR 0.38 95% CI: 0.32–0.44).

## Discussion

Socioeconomic variations in cancer are of interest to inform public health prevention and health care planning, particularly in developing programmes to reduce inequalities in health, for which there are key targets in England [38,38]. While survival and mortality can be influence in the short term cancer incidence requires the latent period before any impacts can be evaluated. Despite this lag time evaluations of cancer, particularly by age, region and deprivation, can assist in public health planning and targeted prevention.

Women in the least deprived socioeconomic groups have higher breast cancer incidence which has been attributed to the association of socioeconomic status with reproductive factors [39] and has been seen in other studies [4,40]. Women from a less deprived socioeconomic group may be more likely to have their first child at a later age, have fewer children in their lifetime and take hormone replacement therapy, with each of these factors associated with increased breast cancer incidence [39-41]. Breast cancer is associated with obesity in post-menopausal women, with the association particularly strong for deprived women [42]. Deprived women have lower levels of screening up-take to the national mammographic screening programme for 50 to 65 year olds (extended to 65 to 69 in 2007) [43-45], however there was little variation by socioeconomic status. The constancy across regions and socioeconomic status may possibly be due to high awareness among all groups. The moderate variations in breast cancer incidence and deprivation gap across regions was consistent with other studies by region and socioeconomic status, with the socioeconomic differences in incidence remaining constant for most regions over time [32]. A Scottish study of inequalities in breast cancer survival found the despite differential screening up-take inequalities in survival had narrowed in the screening ages since the implementation of the screening programme [46].

Cervical cancer screening began nationally in England in 1988 for women aged 20 to 65 years but the age at first screening invitation was raised to 25 years in 2005 [47]. Screening detects dysplastic lesions and *in situ *(CIN III) tumours that are then removed prior to developing into invasive cervical cancer. Most cervical cancers develop as a result of Human Papilloma Virus (HPV), an asymptomatic sexually transmitted infection which is carcinogenic. HPV infection is associated with younger age and higher number of partners [48-50], after adjusting for these factors socioeconomic status was associated with HPV infection in some studies [48,49] while others found no significant association [50]. Screening up-take has been reported to vary by socioeconomic status [51] and be lower for women with low levels of education in England [52]. The lower incidence rates of cervical cancer for Southern England (East, South West, London and South East) are consistent with other national studies [31]. Socioeconomic-variations in screening up-take contribute to the regional variations in deprivation gap, although the regional variations and influence of HPV infection (and associated risk factors) remains an area for further study.

The high lung cancer incidence and deprivation gap in this study is consistent with other studies [31,32]. Smoking is strongly associated with socioeconomic status with over 80% of lung cancer cases are estimated to be attributable to smoking [53,54]. Smoking prevalence rapid decreased during the 1970s and 1980s, with larger decreases occurring in the non-manual groups [55] which is consistent with an increase in the deprivation differences found in other studies [32]. Smoking prevalence in 2006 was higher in the manual occupational groups (27%) than the non-manual groups (17%)[56]. Manual groups were also associated with higher tobacco consumption and beginning smoking at an earlier age [56]. Smoking prevalence by socioeconomic status is not currently available. While occupational and socioeconomic status can not be directly compared evidence suggests that the manual occupational groups are more similar to the more deprived, than the least deprived.

The large deprivation-specific differences found in incidence rates for patients diagnosed with lung cancer under 65, may indicate a cohort effect. In contrast to older ages where rapid decreases in smoking occurred during the 1970s and 1980s there have been larger decreases in the proportion of young men smoking than young women [55,56]. A large cross-sectional study of socioeconomic gradients in smoking among young women in Southampton, England found the prevalence of smoking was highest among the most deprived [57] with the socioeconomic differences increasing with age, as smoking cessation was more common in the least deprived. These trends may suggest a cohort effect with continuing inequalities in lung cancer incidence by age, continued monitoring of smoking prevalence is key to assessing these. In June 2007 a national smoking ban in enclosed public spaces was introduced, it remains to be seen if this will decrease smoking prevalence and/or tobacco consumption.

Incidence of malignant melanoma of the skin in England is expected to increase by 88% in men (66% in women) by 2020 [58], with regional variations in these increases [59]. Regional variations in skin cancer have also been seen in other countries with variations attributed to UV exposure and opportunities for recreational exposure [60-62]. Within the UK the South West receives the most UV exposure [49] and also has an older population with both contributing to the high incidence of malignant melanoma. Exposure to UV has increasingly occurred through sun bed use and holidays abroad which are both significantly associated with malignant melanoma [60]. Higher incidence in the least deprived and regional variations may be largely explained by holidays abroad and exposure to natural UV, however current UV exposure highlights changes to these trends. It is difficult to estimate sun bed use as most are private and unregulated however, anecdotal evidence suggests that sun bed use is increasing in England particularly for teenagers and young adults and may represents a cohort of individuals at higher risk in the future. Exposure to sunbeds before the age of 35 was increased the risk of malignant melanoma by 75% [63]. A study in Dundee, Scotland found the number of sun parlours has increased by up to 30% since 1998 and that 83% of the sun beds exceeded the European standard for UV(B) radiation levels [64]. There is increasing access to sun beds through private unmanned sun parlours, with sun parlours tending to be clustered in areas of deprivation [65]. The cohort of high risk individuals may represent a substantial burden of future skin cancer.

Cancer registries in England collect data to a national specification and have high levels of case ascertainment and consistency [66]. However, there is probably some regional variation due to differences in data collection and timeliness, although this is unlikely to explain the magnitude of differences. Completeness of registration information, such as treatment and stage is known to be less complete for deprived patients [67,68]. These variations would only influence the results of this study if a patients' diagnosis date were changed, and even in this case very little impact would be expected due to grouping of years, however this is unlikely due to the high quality of registry data [66]. Ascertainment of lung and breast cancers are known to be high although [69] regional variations in ascertainment of malignant melanoma have been shown for the early 1990s [31,70,71] with late stage tumours more likely to be registered [72]. Increased use of hospital admission system data in the late 1990s and 2000s would be expected to improved ascertainment, although no direct comparison of this has been published.

Ecological measures of socioeconomic status have been widely used by population-based cancer registries [25,34] and in the absence of individual measures of socioeconomic status are a pragmatic choice. As socioeconomic status is based on area of residence at diagnosis it may not necessarily be representative of every patient's current or historical socioeconomic group and the associated cancer risk factors. However, generally people living in the same area have similar levels of deprivation and have found to be robust over time [34]. This is particularly relevant for cancers where exposure to risk factors occurs years or decades before cancer diagnosis. Alternative measures of socioeconomic status based on individual level data, such as education, occupation or income are also frequently used although cancer registries do not routinely collect these.

## Conclusion

In the long-term decreasing socioeconomic variations in incidence will have a substantial impact on the burden of cancer, for both incidence and mortality. Variations in regional lung and cervical cancer and malignant melanoma rates highlight variations in exposure to risk factors. Lung cancer trends identified larger inequalities in those under 65 at diagnosis, while there were substantial regional and age specific variations for malignant melanoma. Socioeconomic variations in survival can potentially be targeted more quickly while efforts to change incidence will take many years to show any beneficial impact however, in the long term, decreasing inequalities in incidence will also decrease mortality and decrease the number of incident cases.

## Abbreviations

ICD-10: International Classification of Diseases version 10; LSOA: lower super output areas; IMD: Index of Multiple Deprivation; GOR: Government Office Region; HPV: Human Papilloma Virus; CIN III: Carcinoma *in situ*; UV: Ultra Violet Radiation.

## Competing interests

The authors declare that they have no competing interests.

## Authors' contributions

CJ, CT, LS, HM and VM were involved in the study design with the statistical analysis carried out by CJ and LS. LS wrote the draft of the manuscript to which all authors subsequently contributed. All authors contributed to the interpretation of results and revision and approval of the final manuscript.

## Pre-publication history

The pre-publication history for this paper can be accessed here:


